# Treatment of Cesarean Scar Ectopic Pregnancy Using a Double Balloon System

**DOI:** 10.1097/og9.0000000000000020

**Published:** 2024-07-11

**Authors:** Jaclynne Hedge, Jeannie Kelly, Ashley Veade, Shelby Dickison

**Affiliations:** Department of Obstetrics and Gynecology and the Division of Maternal Fetal Medicine, Washington University School of Medicine in St. Louis, St. Louis, Missouri.

## Abstract

Cesarean scar pregnancies are increasing in incidence, with ill-defined management options. We applied a newly published cesarean scar ectopic pregnancy clinical classification system to our previously published, now expanded, case series of cervical double-balloon catheter management to inform this option as a potential surgical treatment strategy. We found that cervical double-balloon catheter management of cesarean scar pregnancies is an efficacious and safe option when utilizing the newly published clinical classification system. Of our 34 recorded cases, only 5.9% (2/34) of patients required additional procedures after initial cervical double-balloon catheter. Cervical double-balloon catheter should be considered as potential management in future classification systems.

## Abstract 2

Cervical double balloon catheter management of cesarean scar ectopic pregnancies is comparable with other management options in a new classification system and should be considered when counseling patients.

Cesarean scar pregnancies can result in severe morbidity and are increasing in incidence. Current reported treatments are limited by a predominance of case series; the optimal management is unknown, and complications after treatment have been reported to be as high as 44%.^1^ A minimally invasive option using a cervical double-balloon catheter was first described by Timor-Tritsch et al^2,3^ as a highly effective option with a low complication rate, and our institutional experience demonstrated similar findings.^4^ Ban et al^5^ published a new classification system that informed surgical management options based on myometrial thickness (mm) and average diameter of the gestational sac (mm) but did not consider cervical double-balloon catheter management. We sought to explore how the Ban classification system affected outcomes for cesarean scar pregnancies managed using the cervical double-balloon catheter at our institution.

## METHODS

From October 2018 to February 2024, patients diagnosed with cesarean scar pregnancies were counseled on management options, including cervical double-balloon catheter. Those who elected for cervical double-balloon catheter management underwent the procedure following previously published guidelines.^2^ The protocol for cervical double-balloon catheter placement at our institution is as follows: 1) positioning patient into the lithotomy position, a speculum is inserted and the cervix is prepped with betadine or chlorhexidine; 2) a tenaculum is placed on the anterior lip of the cervix, and the cervix then is dilated to a Heger 7 or 21 Pratt under ultrasonographic guidance transabdominally to avoid disrupting the cesarean scar pregnancy; 3) a cervical double-balloon catheter with a stylet then is inserted to the fundus using a ring forceps under ultrasonographic guidance, and 10 mL of sterile saline is injected to secure the upper balloon; 4) the speculum and tenaculum then are removed, and an additional 10 mL of sterile saline is placed in the lower balloon; 5) sterile saline then is added progressively to the upper and lower balloons, based on patient comfort, to compress the gestational sac. The amount of fluid varied to from 5 mL to 40 mL in the upper balloon and 0 mL to 50 mL in the lower balloon. This variation in fluid amount in the upper and lower balloons represents the varying amount of fluid needed to achieve appropriate compression of the gestational sac based on gestational age, patient comfort, and clinician discretion. Repeat ultrasonography is performed to confirm placement. Starting in July 2019, in response to updated guidelines, a single dose of 50 mg/M^2^ of adjuvant intramuscular methotrexate is also given.^1^ During the time of placement, no specific analgesia regimen is routinely offered, because this procedure is well-tolerated. However, based on clinician and patient discretion, some patients received cervical blocks with lidocaine with epinephrine, intravenous 1-mg hydromorphone, or 25 micrograms of fentanyl. Afterward, patients are encouraged to take ibuprofen as needed for pain.

Patients were tracked prospectively in a quality-improvement database, collecting patient demographics, β-hCG levels, pregnancy outcomes, and clinician experience with the procedure. In 2023, all cases were classified using the Ban^5^ system at entry or by retrospective review of ultrasound imaging and measurement of anterior myometrial thickness overlying the gestational sac by a trained clinician. Cesarean scar pregnancies with anterior myometrial thickness greater than 3 mm were classified as type I, those with anterior myometrial thickness between 1 and 3 mm with gestational sac 30 mm or less were classified as type IIA, those with anterior myometrial thickness between 1 and 3 mm with gestational sac larger than 30 mm were classified as type IIB, those with anterior myometrial thickness less than 1 mm with mean gestational sac diameter less than 50 mm were classified as type IIIA, and those with anterior myometrial thickness less than 1 mm with mean gestational sac diameter greater than 50 mm were classified as type IIIb.^5^ Primary outcomes were pregnancy resolution rates and maternal complication rates for patients with type I, II, and III cesarean scar pregnancies treated with cervical double-balloon catheter management. We additionally compared the rate of β-hCG resolution and need for further procedures among groups. Based on the quality-improvement nature of this study, it was IRB-exempt.

## RESULTS

Of 34 cesarean scar pregnancies treated with cervical double-balloon catheter placement, 11 (32.3%) were type I, 19 (55.8%) were type IIA, and four (11.8%) were type IIIA based on their anterior myometrial thickness. Thirty (88.2%) of the patients with cesarean scar pregnancies included in the new classification system received adjuvant systemic methotrexate based on timing of treatment.

There were no differences in patient demographics or pregnancy characteristics in each category of cesarean scar pregnancy (Table 1). Race was analyzed to reflect our diverse patient population to make the findings more generalizable. Nine patients were lost to follow-up—three with type I cesarean scar pregnancies, three with type II, and three with type III. Twenty-six patients had β-hCG levels followed to 0. The average time to β-hCG resolution was 48.5 days for all patients with cesarean scar pregnancies and varied by type of cesarean scar pregnancy. All pregnant patients were successfully treated with cervical double-balloon catheter placement, including two with dichorionic diamniotic twin gestations (type I and type IIIA).

**Table 1.** Patient Characteristics

Characteristic		Cesarean Scar Pregnancy Type			
		Type I (n=11)		Type II (A and B) (n=19)	Type III (A and B) (n=4)
Age (y)		33.2±6.05 32 [26, 44]		33.4±4.6 34 [26, 44]	34.2±4.9 34.5 [29, 39]
Race					
Asian		0 (0)		2 (10.5)	0 (0)
Black or African American		5 (45.5)		7 (36.9)	1 (25)
White		6 (54.5)		10 (52.6)	3 (75)
BMI (kg/m^2^)		32.2±9.4 29 [22, 49]		33.4±6.09 32 [22, 43]	31.5±7.9 30.5 [24, 41]
Gestational age at presentation (d)		45.9±7.7 44 [35, 64]		48.8±8.6 45 [38, 71]	50±5.4 49.5 [44, 57]
Initial β-hCG level (milli-international units/mL)		35,501.3±37,540.3 29,709 [1,938, 123,532]		31,932±36,595.8 16,645 [1,409, 136,746]	26,852±24,073.0 24,801 [2,936, 54,870]
Parity		2.63±1.3		2.74±1.3	2±0.8

BMI, body mass index.

Data are mean±SD, median [minimum, maximum], or n (%).

There were no cases of maternal hemorrhage requiring transfusion, hysterectomy, or intensive care unit admission for any type of cesarean scar pregnancy (Table 2). Two patients required additional procedures, as mentioned in Table 2.

**Table 2.** Cesarean Scar Pregnancy Outcomes After Cervical Double-Balloon Catheter Placement

Patient Number	Gravidity	Parity	BMI (kg/m^2^)	GA at Diagnosis	No. of Prior Cesareans	No. of Prior CSPs	FHM	Initial β-hCG Level (Milli-International Units/mL)	Anterior Myometrial Thickness (mm)	Mean Sac Diameter (mm)	CSP Type (New System)	Days Catheter in Place	Balloon Fluid (mL)	MTX	Days to Documented β-hCG Resolution	Complications	Comments
Type I																	
1	9	3	47	7.0	3	0	Yes	29,709	5	17.6	I	2	25/25	Yes	35		
2	1	0	24	5.0	0	0	No	1,938	3.1	4.4	I	1	15/10	No	20		
3	6	3	49	7.3	3	0	Yes	76,277	3.7	12.6	I	3	40/40	Yes	83		
4	8	3	23	6.2	3	0	Yes	5,157	5.4	NF	I	2	7/0 (Foley)	Yes	26		Single Foley balloon used due to supply issues during COVID-19 pandemic
5	2	1	22	6.6	1	0	No	33,665	3.4	6.2	I	2	15/10	Yes	103		
6	5	3	28	6.2	3	0	No	20,427	4.5	16	I	2	35/35	Yes	LTFU		Subsequent dichorionic diamniotic twin IUP
7	6	4	25	6.3	4	0	Yes	56,639	3.7	3.9	I	3	30/20	Yes	LTFU		Resumption of regular menses
8	6	4	36	9.1	4	0	Yes	123,532	10.7	17.3	I	2	40/15	Yes	LTFU		Required additional MTX dose, D&C, and dx lsc
9	3	2	29	6.1	2	0	Yes	33,957	3.4	5.4	I	3	15/15	Yes	43		
10	4	2	37	5.2	2	0	No	2,248	3.1	14	I	2	15/15	Yes	35		
11	7	4	34	6.2	2	0	No	6,966	3.2	12.5	I	2	10/0	Yes	180		LTFU for several months; β-hCG negative before tubal ligation
Type II																	
12	4	2	24	8.0	2	0	Yes	136,746	1.8	10	IIA	2	40/50	No	93	AVM, embolized 5 mo later	
13	4	1	32	6.0	1	0	Yes	9,909	2	2.0	IIA	3	15/20	No	80		Subsequent IUP
14	11	4	32	8.1	4	0	No	1,409	2.3	3.4	IIA	2	30/0	Yes	22		
15	4	2	22	8.1	1	0	No	3,182	1.2	7.5	IIA	1	8/8	Yes	31		Subsequent IUP
16	5	2	39	8.1	3	0	No	19,703	2	16	IIA	1	25/25	Yes	22	Fever, UTI on day 7, treated with antibiotics	
17	11	5	36	6.1	4	0	Yes	14,515	3	4.5	IIA	3	20/15	Yes	29		
18	4	2	30	8.3	2	0	No	21,523	3	20	IIA	1	15/13	Yes	56		
19	4	3	37	6.3	3	0	No	13,716	2.8	34	IIA	2	25/15	Yes	31		
20	9	4	29	5.3	4	0	Yes	9,828	2.2	9.1	IIA	1	28/30	Yes	23		
21	4	2	43	6.2	2	0	No	2,295	2	8.0	IIA	2	30/10	No	16		
22	4	2	34	5.3	2	0	No	11,872	2.7	8.4	IIA	1	5/10	Yes	LTFU		
23	5	1	39	6.6	1	0	No	37,156	2	1.9	IIA	2	15/20	Yes	23		
24	3	2	24	6.2	2	0	Yes	37,638	2.4	5.4	IIA	1	20/15	Yes	59		
25	4	3	30	10.1	3	0	Yes	65,415	2	3.37	IIA	2	35/40	Yes	60		
26	4	1	37	6.1	1	0	Yes	44,187	2.5	4.4	IIA	2	35/25	Yes	79		
27	6	5	32	7.2	5	0	Yes	47,083	3	10.3	IIA	2	30/15	Yes	LTFU		
28	7	4	39	6.1	4	0	No	6,110	3	23	IIA	2	10/40	Yes	43		
29	7	4	43	6.1	4	0	No	16,645	1.1	12.5	IIA	2	10/15	Yes	35		
30	4	3	32	7.0	3	0	Yes	107,786	2.7	9.6	IIA	2	15/7	Yes	LTFU		
Type III																	
31	3	2	41	7.1	2	0	Yes	11,142	1	10.2	IIIA	1	30/50	No	30		
32	6	3	26	6.2	3	0	Yes	38,460	1.7	0	IIIA	2	30/18	Yes	LTFU	Dichorionic diamniotic twin CSP treated	
33	3	1	24	8.1	1	0	Yes	54,870	0	20.2	IIIA	2	15/15	Yes	LTFU		
34	3	2	35	7.0	2	0	No	2,936	0	NF	IIIA	2	17/25	Yes	LTFU		

BMI, body mass index; GA, gestational age; CSP, cesarean scar pregnancy; FHM, fetal heart motion; MTX, methotrexate; NF, not found; COVID-19, coronavirus disease 2019; LTFU, lost to follow-up; IUP, intrauterine pregnancy; D&C, dilation and curettage; dx lsc, diagnostic laparoscopy; AVM, arteriovenous malformation; UTI, urinary tract infection.

## DISCUSSION

Recent studies have created a new classification system for cesarean scar pregnancy for which cervical double-balloon catheter has not been described as a treatment option. Our data offer preliminary evidence that cervical double-balloon catheter with systemic methotrexate is a successful, nonsurgical, and fertility-sparing treatment option for type I, type IIA, and type IIIA cesarean scar pregnancies, including twin gestations, without an increase in maternal morbidity. We had only two patients who required further interventions, but neither required hysterectomy or blood transfusion. Our time to β-hCG resolution was more than two times as long as that in Ban et al^5^ (mean 48.5 days vs 21 days), although it is unclear from their data set whether there were specific management options that increased the rate of decline. We also had nine patients who were lost to follow-up and four patients who did not originally receive methotrexate when we began our project, which could be affecting this number. This study is limited by the small number of cases of cesarean scar pregnancy currently, and further investigation is needed regarding the efficacy of cervical double-balloon catheter placement over other treatment strategies in the new classification system.
